# Reproducibility of Variant Calls in Replicate Next Generation Sequencing Experiments

**DOI:** 10.1371/journal.pone.0119230

**Published:** 2015-07-02

**Authors:** Yuan Qi, Xiuping Liu, Chang-gong Liu, Bailing Wang, Kenneth R. Hess, W. Fraser Symmans, Weiwei Shi, Lajos Pusztai

**Affiliations:** 1 Departments of Bioinformatics and Computational Biology, University of Texas MD Anderson Cancer Center, Houston, Texas, United States of America; 2 Department of Experimental Therapeutics, University of Texas MD Anderson Cancer Center, Houston, Texas, United States of America; 3 Department of Breast Medical Oncology, University of Texas MD Anderson Cancer Center, Houston, Texas, United States of America; 4 Department of Biostatistics, University of Texas MD Anderson Cancer Center, Houston, Texas, United States of America; 5 Department of Pathology, University of Texas MD Anderson Cancer Center, Houston, Texas, United States of America; 6 Yale Cancer Center, Yale School of Medicine, New Haven, Connecticut, United States of America; Ecole normale superieure de Lyon, FRANCE

## Abstract

Nucleotide alterations detected by next generation sequencing are not always true biological changes but could represent sequencing errors. Even highly accurate methods can yield substantial error rates when applied to millions of nucleotides. In this study, we examined the reproducibility of nucleotide variant calls in replicate sequencing experiments of the same genomic DNA. We performed targeted sequencing of all known human protein kinase genes (kinome) (~3.2 Mb) using the SOLiD v4 platform. Seventeen breast cancer samples were sequenced in duplicate (n=14) or triplicate (n=3) to assess concordance of all calls and single nucleotide variant (SNV) calls. The concordance rates over the entire sequenced region were >99.99%, while the concordance rates for SNVs were 54.3-75.5%. There was substantial variation in basic sequencing metrics from experiment to experiment. The type of nucleotide substitution and genomic location of the variant had little impact on concordance but concordance increased with coverage level, variant allele count (VAC), variant allele frequency (VAF), variant allele quality and p-value of SNV-call. The most important determinants of concordance were VAC and VAF. Even using the highest stringency of QC metrics the reproducibility of SNV calls was around 80% suggesting that erroneous variant calling can be as high as 20-40% in a single experiment. The sequence data have been deposited into the European Genome-phenome Archive (EGA) with accession number EGAS00001000826.

## Introduction

Massively parallel, next generation sequencing is increasingly used to identify nucleotide alterations in the human genome including single nucleotide variants (SNV) and indels. Several studies have compared the performance of various next generation sequencing platforms [1] and enrichment methods [2, 3] to one another and showed that each method has unique advantages and disadvantages but their overall performances are similar. So far, few studies examined the technical reproducibility of sequencing findings in repeat measurements. The accuracy of next generation sequencing results is often gauged by their ability to detect known polymorphisms in a given sample. Most methods show > 95% sensitivity for detecting SNVs. While sensitivity is an important metric, it does not capture all important aspects of assay performance including overall accuracy and specificity. Specificity is particularly relevant because these methods are often employed not only to detect known polymorphisms, which could be done more cost effectively with other methods, but to find novel sequence variants. Any observed SNV may represent a true biological variant or it may be a technical error. Even highly accurate and sensitive sequencing methods can yield large numbers of erroneous calls when applied to billions of nucleotides (e.g. a method that misidentifies 1 out of 10,000 nucleotides would yields around 30,000 false calls over the 3 billion measurements of the human genome). Since the number of true variants in a sample is never known and the magnitude of technical noise in next generation sequencing methods is not well defined, the signal to noise ratio of the results and probability of false discovery remains largely unknown and may be underreported.

When aliquots of the same source DNA are analyzed twice, discordant results represent a measure of technical noise. In this study, we examined the reproducibility of nucleotide variant calls in replicate sequencing experiments including library preparation and target region capture of the same starting genomic DNA. As kinases are major cancer drug targets, we performed targeted sequencing of all known human protein kinase genes (kinome). Genomic DNA from 17 different breast cancer samples were sequenced in duplicate (n = 14) or triplicate (n = 3) to assess overall concordance of all nucleotide calls and variant calls. We also examined what factors influence concordance rates including variations in DNA quality and quantity (above a quality control threshold), length of DNA storage, the type of nucleotide substitution, the genomic location of the variant, the depth of coverage and various matrices of the variant call itself (e.g. allele count, allele frequency).

## Material and Methods

### Ethics Statement

This study was approved by the Institutional Review Board of the University of Texas MD Anderson Cancer Center. All patients have signed informed consent to undergo a research biopsy and for genomic analysis of their cancer.

### Patient samples

Fine needle aspiration samples of newly diagnosed stage I-III (n = 12) and metastatic (n = 5) breast cancers were obtained in the context of a series of biomarker discovery studies at the University of Texas MD Anderson Cancer Center between 1999–2010. This study was approved by the Institutional Review Board. Cells from 2 needle passes were collected into a vial containing 1 ml of RNAlater solution (Ambion, Austin, Texas) and were stored at– 80°C until total RNA and DNA extraction. DNA was extracted with the QIAamp DNA Mini Kit (Qiagen) following the manufactures instructions from the stored flow-through part of a preceding RNA extraction step (RNeasy mini kits, Qiagen, Valencia, California). Final DNA concentration and purity were assessed by Nanodrop 2000 Spectrophotometer (Thermo Fisher Scientific Inc., Pittsburgh, Pennsylvania) and Agilent Bioanalyzer 2100 (Agilent Technologies, Wilmington, Delaware). Sequencing was performed if at least 1.8 μg genomic DNA could be recovered and if DNA 260/280 OD ratio was > 1.5. Aliquots of the same starting DNA were sequenced in duplicates (n = 14) and triplicates (n = 3) including complete replication of the genomic library preparation, target capture, amplification and sequencing. Each sample had its unique barcode as part of a 16-sample multiplexed library per SOLiD sequencing slide. The 37 replicate samples (14x2 + 3x3) were sequenced in seven different batches (i.e. seven different slides) from October 2010 to April 2011 (S1 Table).

### Kinome capture and SOLiD sequencing

#### Kinome sequencing library preparation

The SureSelect XT Target Enrichment Kits for AB SOLiD Multiplexed Sequencing (Version 1.0, Nov.2010) was used for kinome sequencing library preparation. In brief, 1.8–3.0 μg of genomic DNA from each sample was fragmented into peak fragment size of 150–180 base pairs (Covaris S2 instrument, Covaris Inc. Woburn, Massachusetts), fragment purification was performed with Agenecourt AMPure plus beads (Beckman, P/N A63881) followed by end repair using T4 DNA polymerase and Klenow DNA polymerase at room temperature for 30 minutes. The purified, end-repaired fragments were ligated with P1 and 1A adaptors on both ends at room temperature for 15 minutes. Subsequently, 200bp DNA fragments with ligated adaptors were isolated by electrophoresis using E-gel SizeSelect 2% gel (Invitrogen, P/N G661002) and were amplified by nick translation performed on PCR 9700 thermocycler using SureSelect pre-capture primers x 12 cycles. The PCR products were purified and quantified by Agilent bioanalyzer 2100 DNA 1000 assay. The expected size distribution of this amplified genomic DNA library with P1 and truncated multiplex P2 adaptors is 250–275bp.

#### Target enrichment

Target enrichement was performed individually by using the Agilent SureSelect Human Kinome XT Kit and Target Enrichment system that targets 3.2 Mb of the human genome including all known human kinases and a selected group of other cancer-related genes and their associated untranslational regions (www.agilent.com/genomics/sureselect). Five hundred ng of DNA from individual genomic libraries was hybridized with SureSelect kinome capture library, which is a mixture of 120 nucleotide-long biotinylated RNA baits used as probes for 612 target genes designed from 10,282 exons, following the manufacturer's instructions. After 24 hours of hybridization at 65°C, the target regions were isolated by pulling down the biotinylated probe/target hybrids with streptavidin-coated magnetic beads (Dynal MyOne Streptavidin T1, Invitrogen). The captured target regions were purified using Agenecourt AMPure beads. The target DNA libraries from each replicate were enriched and multiplexing barcodes were added through a 9-cycle PCR amplification step using the SureSelect SOLiD barcode multiplexing PCR primer set. The PCR products representing the final individual, enriched, kinome sequencing library were purified by Agenecourt AMPure Plus beads and quantitated with Agilent Bioanalyzer 2100; the expected size distribution of the final target library is 270–350 base pairs.

#### Construction of multiplexing libraries

SOLiD paired end sequencing kinome multiplexing libraries were constructed from the equal pooled, individual, barcoded kinome library above. Batches of 16 barcoded, individual kinome libraries were pooled in equal molar ratio as one multiplexed library and sequenced in one session. This analysis focuses on 37 replicate libraries that were part of a larger sequencing project including a total of 112 libraries. A total of seven pooled, multiplexed libraries were sequenced (7x16 = 112) on seven separate dates; each sequencing batch included at least 2 replicate samples from another batch. The 600–700 million template beads generated from each multiplexing library by emulsion PCR, corresponding to the full length templates containing both P1 and P2 adaptors, were further modified by adding oligo-linkers to immobilize template beads and deposited on a pre-coated glass slide that was loaded onto the flow cell of SOLiD V4 Genome Analyzer (Life Technologies, Carlsbad, CA). The barcoded, multiplex, paired end (5+35+50 nucleotides) sequencing run of each slide was performed following the SOLiD operation instructions.

### Sequencing data analysis

#### Sequence analysis pipelines

All sequenced reads in paired end of 50 (F3-tagged reads) and 35 (F5-tagged reads) nucleotides on the same sequencing templates in color-space from each barcoded individual sample were processed by the BioScope software. The data on quality metrics (*.qual files) and color calls (*.csfasta files) were fed into Applied Biosystems BioScope software (version 1.3). We used the software to carry out the following modular data analyses sequentially, including applying the SOLiD Accuracy Enhancer Tool (SAET) to F3- and F5-tagged reads, mapping of the F3 and F5 reads, paired-end pairing of F3 and F5 reads, enrichment on the target regions, and error reporting of each read position. The results were then used to detect (“call”) SNVs using the diBayes models in BioScope. Based on the length of the target region and the size of libraries, the estimated average coverage was greater than 80x; therefore, we set the SNV-call stringency parameter to the highest level in BioScope. The reference genome used in this analysis was GRCh37 (hg19). Spearman and concordance correlation coefficients of the basic sequencing metrics were calculated for the paired replicates. Coverage and genotype of the entire sequenced regions were taken from BioScope output files *_Consensus_Calls.txt for each sample. The number of total F3-tagged reads was used as the number of total reads of the pair of F3- and F5-tagged reads. Statistical analysis of the data generated by BioScope was carried out in statistical language R. The sequence data have been deposited into the European Genome-phenome Archive (EGA) with accession number EGAS00001000826.

The ANNOVAR software [4] was used to annotate the observed SNVs based on their genomic locations. The upstream and downstream annotations refer to variant overlapped 1-kb region upstream or downstream of transcription start site or end site, respectively. The Mutation Assessor (MA) [5] was used to predict the functional importance of SNVs. The Mutation Assessor scores and prediction categories were obtained though MA’s web-based application programming interface (http://mutationassessor.org/). High functional importance (HFI) SNV was defined as either (i) predicted to be in the high or medium functional importance category by the Mutation Assessor, or (ii) a stopgain (nonsense) or (iii) stoploss variant.

Duplicated reads removal was carried out using software Picard MarkDuplicates (http://broadinstitute.github.io/picard). VarScan2 SNV calls were generated and filtered by using SAMtools [6] mpileup and VarScan2 [7].

#### Concordance rate calculation

The concordance rate, *R*_*c*_, between duplicate samples was defined as

$$R_{c}=\frac{N_{c}}{mean(N_{1},N_{2})}$$

where *N*_*c*_ was the number of concordant SNVs between a pair of replicate samples (i.e., variants that were detected in both samples), and *N*_*1*_ and *N*_*2*_ were the total number of SNVs detected in each of the duplicated sample (i.e., the sum of concordant and discordant NVs). For triplicate measurements, three pairwise concordance rates among the replicates were calculated, and their average was used as the concordance rate for that case.

We examined concordance rate by seven different factors and the values of each factor were divided into different categories (i.e. bins), based on (i) natural categories, e.g. nucleotide substitution type (A->C, A->G, C->T, *etc*.), and gene annotation type (exonic, intronic, *etc*.); (ii) empirical values, e.g. coverage was divided into five categories: [1,5), [5,20), [20,80), [80,200), ≥200, or (iii) algorithmically generated bins using Sturges' formula that was implemented in the histogram function in R. These bins were generated by using the values of all of the 37,268 SNV positions that were detected in the 37 samples. Bins at the two tails that contained very small number of positions were combined together. Factors whose categories were generated using this method included variant allele (also called non-reference allele or novel allele) count, variant allele frequency, variant allele quality, and SNV-call p-value. The histogram bins for variant allele count was generated using log10-transformed variant allele count values. The concordance rate by a factor was calculated within each category of the factor. The factor values at every position in the entire sequenced regions were taken from BioScope output files *_Consensus_Calls.txt for each sample. For a pair of replicated samples, only chromosomal positions in both samples that were in the same category of a factor were used to calculate concordance rate for that factor. Positions that were in different categories for a factor in the paired samples were disregarded for concordance rate calculation of that factor. Wilcoxon rank sum test or two-sided t-test was used to test the significance of the difference in concordance rates in different categories.

#### Association tests between factors and concordance status

Concordance status was coded as 1 (concordant) or 0 (discordant) for a chromosomal position in a pair of replicated samples. Three methods were used to test the strength of association between a factor and concordance status, including mutual information, AIC, and Lasso regression. Mutual information between a factor and the concordance status was calculated by treating the factor as a discrete variable using the same bins that were used in calculating concordance rate. Akaike information criterion (AIC) between a factor and the concordance status was calculated using univariate logistic regression. Multivariate Lasso logistic regression was performed using R package glmnet [8] with default setting. Both AIC calculation and Lasso regression analysis were performed by using the factors as continuous variables.

## Results

### Variations in basic sequencing metrics in replicate experiments

**Fig 1:**
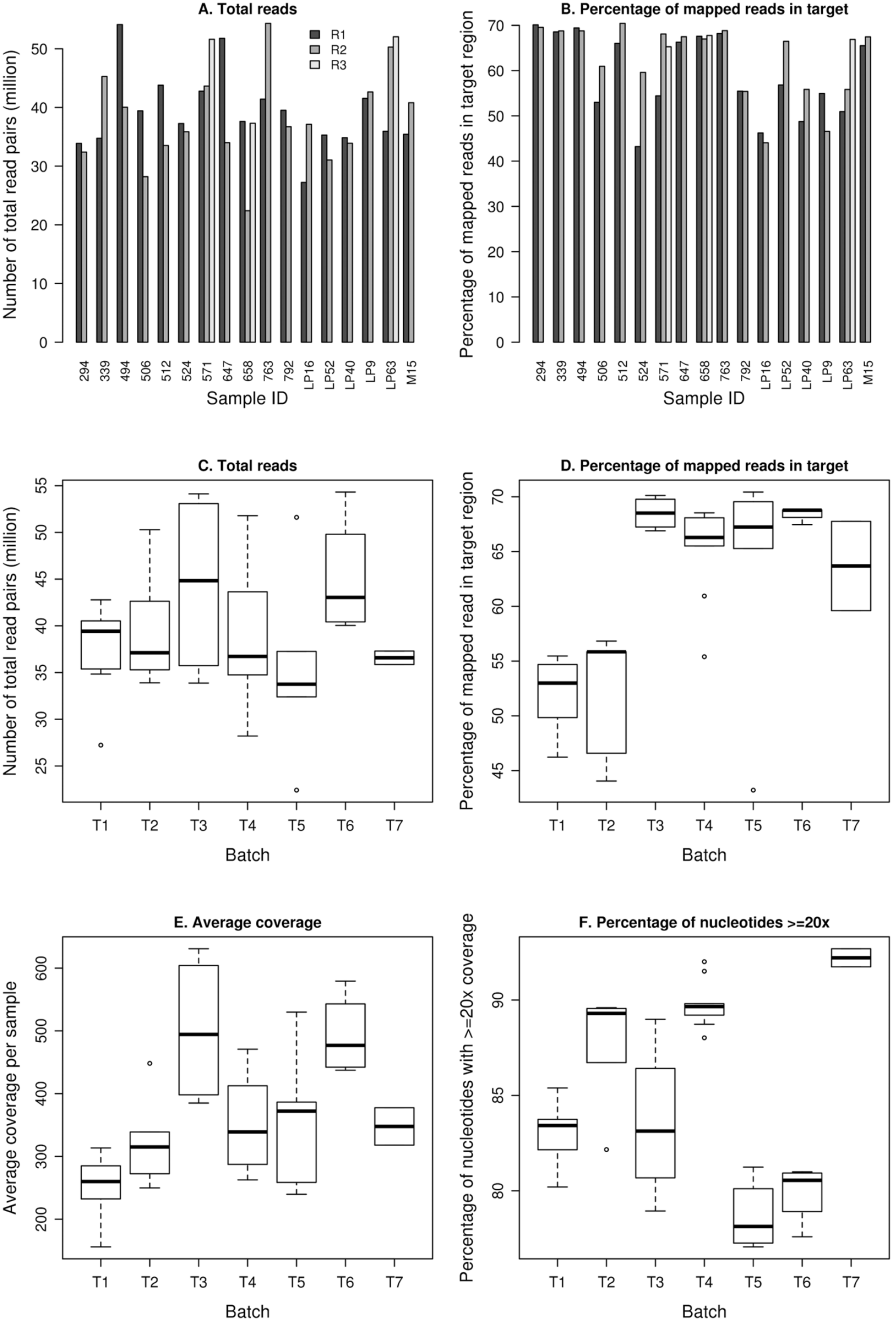
Barplots and boxplots showing variations in basic sequencing metrics between replicated samples and batches. Barplots of (A) the number of total reads (i.e. number of reads of F3- and F5-tagged paired reads), and (B) percentage of mapped reads in target region for each of the replicate pairs. The boxplots show the batch-to-batch differences in the number of total reads (C), the percentage of mapped reads in the target region (D), the average coverage (E), and the percentage of nucleic acids with ≥20x coverage within the target region (F).

The basic sequencing metrics varied between samples and replicated experiments. The number or total pairs of reads in the 37 experiments ranged from 22.4 to 54.3 million and the percentage of reads that mapped to targeted regions, defined as regions in the BED file provided by Agilent as their designed target, ranged from 43.22% to 70.43%. Fig 1A and 1B show bar graphs of the number of read pairs (i.e. number of F3- and F5-tagged paired reads) and the percentage in target region for each of the replicated samples. Coverage depth (i.e. number of the reads at a given nucleotide position) ranged from 0 to 32,100 for individual nucleotide positions and the average coverage depth ranged from 156 to 631 across the 37 experiments. Between each of the seven sequencing batches, the number of total read pairs, the percentage of reads mapped to a targeted region, the average depth of coverage, and the percentage of nucleotides with >20x coverage in the target region all showed relatively large variations (Fig 1C–1F). Table 1 shows the percentage of nucleotides in the targeted region with >1x and >20x coverage, respectively, for each replicate. The percent of nucleotides in the targeted region with >20x coverage ranged from 77–93% in individual experiments. The Pearson correlation coefficients for coverage depth for all nucleotide positions that had at least one read in both replicate pairs ranged from 0.29 to 0.77.

**Table 1:** The percentage of target base pairs with at least 1x or 20x coverage for each replicated sample.

	≥1x Coverage			≥20x Coverage		
Sample ID	R1	R2	R3	R1	R2	R3
294	95.99	93.27		88.99	77.26	
339	95.87	94.79		88.73	80.87	
494	94.22	94.67		83.84	80.99	
506	93.86	96.46		80.2	89.79	
512	95.29	93.65		88.02	78.88	
524	93.73	97.12		77.06	92.69	
571	96.11	95.64	94.69	85.39	89.66	81.24
647	96.43	94.31		92.01	80.11	
658	92.57	93.76	96.39	78.94	77.38	91.74
763	95.94	92.77		89.81	77.59	
792	94.06	96.17		81.32	89.21	
LP16	95.99	96.49		82.98	86.72	
LP52	96.79	96.23		89.56	91.51	
LP40	95.97	97.01		83.42	89.3	
LP9	95.46	94.03		84.03	82.16	
LP63	95.63	96.82	94.05	83.46	89.6	82.42
M15	95.65	94.29		89.31	80.23	

R1 = sequencing experiment 1, R2 = sequencing experiment 2, R3 = sequencing experiment 3.

### Concordance over all nucleotide calls

To verify the overall reproducibility over the entire sequenced target regions, we calculated the concordance rate of all nucleotide calls. The concordance rates over the entire covered sequenced regions ranged from 97.0% to 99.0%. The majority of discordant calls were due to ambiguous calls where one call of a pair was a specific nucleotide (A, T, C, or G) and the other call of the pair was "N" (which stands for any of the four nucleotides, equivalent as missing). Such ambiguous calls composed of 1–3% of the entire sequenced regions. If we consider only unambiguous calls, the overall concordance rates increased to 99.99% (Table 2), which is in agreement with the low technical error rates in nucleotide calls claimed by manufacturers.

**Table 2:** Concordance rates between pairs of replicated samples of all unambiguous nucleotide calls over the entire sequenced regions and of SNV calls.

Sample ID	Replicate type	Concordance rate of all unambiguous nucleotide calls (N_cc/N_total)	Concordance rate of SNV calls (N_cc/N_total)
294	Dup	99.9919% (3402302/3402578)	64.4% (567/880)
339	Dup	99.9902% (3531773/3532119)	68.2% (634/930)
494	Dup	99.9899% (3527458/3527813)	68.1% (719/1056)
506	Dup	99.9905% (3260377/3260687)	52.7% (430/816)
512	Dup	99.9914% (3244079/3244357)	59.5% (412/692)
524	Dup	99.9915% (3596181/3596485)	71.8% (851/1186)
647	Dup	99.9917% (3554321/3554617)	66.9% (615/919)
763	Dup	99.9938% (3218631/3218829)	61.2% (394/643)
792	Dup	99.9909% (3301138/3301440)	56.2% (461/820)
LP16	Dup	99.9910% (3584535/3584858)	72.8% (846/1162)
LP52	Dup	99.9865% (3667521/3668018)	64.2% (803/1251)
LP40	Dup	99.9880% (3625547/3625983)	71.4% (989/1386)
LP9	Dup	99.9947% (3220037/3220209)	61.6% (414/672)
M15	Dup	99.9908% (3488692/3489012)	65.9% (558/846)
571	Trp	99.9866% (3448705/3449176)	57.7% (465/905)
		(3548364/3548819)	(813/1186)
		(3418099/3418572)	(487/916)
658	Trp	99.9938% (3417223/3417415)	72.2% (748/1024)
		(3497328/3497561)	(760/1083)
		(3548737/3548960)	(816/1108)
LP63	Trp	99.9929% (3537753/3538017)	70.3% (660/946)
		(3467992/3468237)	(636/912)
		(3510910/3511142)	(692/969)

Replicate Type refers to duplicated (Dup) or triplicated (Trp) measurements. In parenthesis after the concordance rates are the actual numbers of concordant nucleotide positions (N_cc) and the average total number of sequenced nucleotide positions or SNV calls in the pair (N_total). For triplicated samples, the concordance rates shown are the averages of the concordance rates across the three pairs of measurements.

The goal of sequencing experiments is often to find novel or different SNVs between two genomes; therefore, we next examined the reproducibility of variant calls. The average number of SNV calls per sample was 961. When defining concordance of SNV calls between a pair of replicated samples, genotype comparison was used. For all SNV calls, the concordance rate per replicated sample ranged from 52.7% to 72.8%, with a median of 68.1% (Table 2). For high functional important (HFI) SNVs, the concordance rate per replicated sample ranged from 0% to 75.0%, with a median of 57.1%. These low concordance rates indicate substantial technical noise in the variant calls suggesting that only a fraction of them represent true variants.

### Factors that affect concordance of variant calls

In order to identify factors that influence reproducibility of variant calls, we examined concordance rates by different biologically and technical factors including nucleotide substitution type, genome annotation type, coverage, variant allele count (VAC), variant allele frequency (VAF), variant allele mapping quality, SNV call p-value, and GC content.

**Fig 2:**
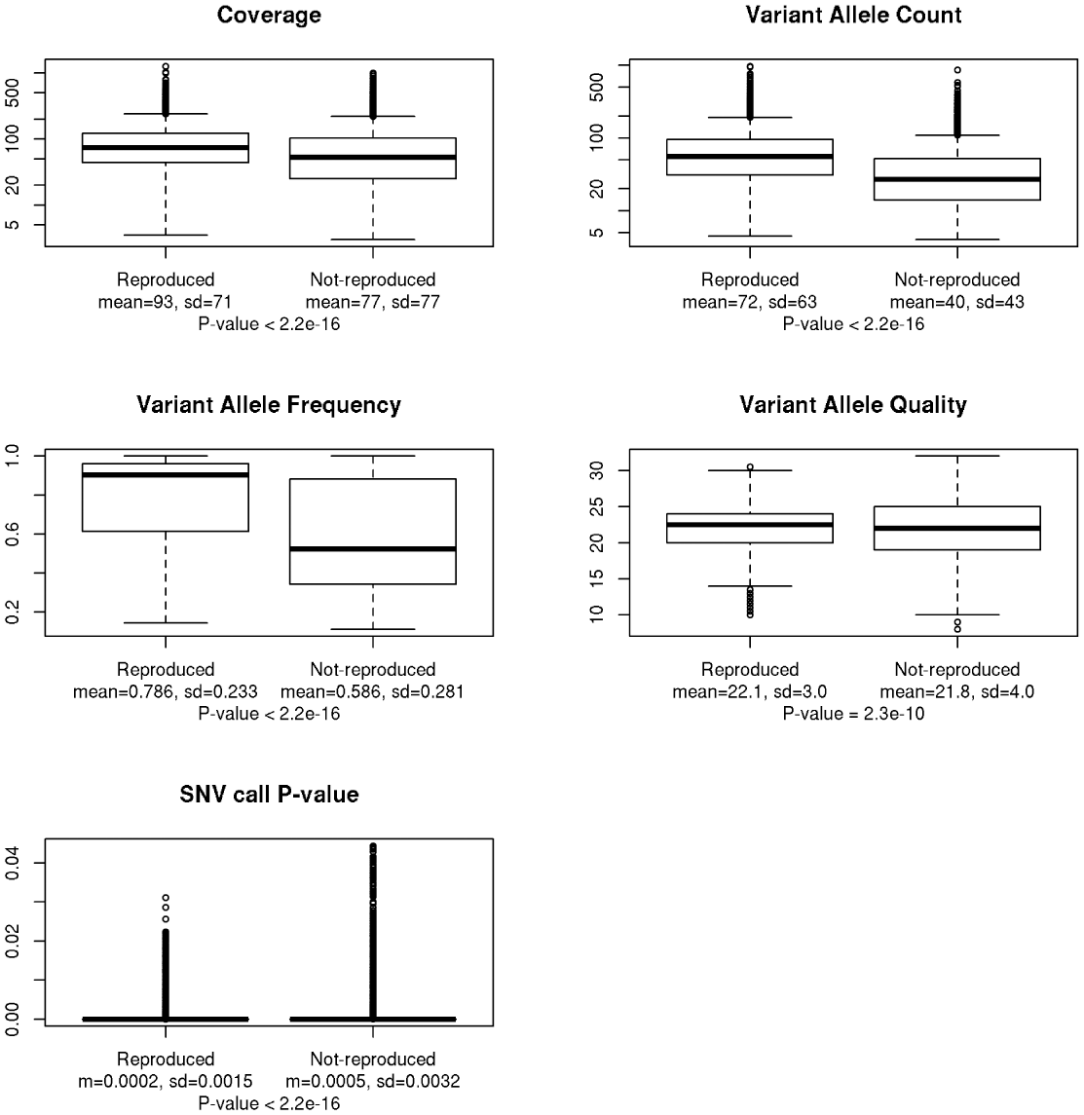
Boxplots comparing the values of different factors between the pooled reproduced and not-reproduced SNV calls. The factors include coverage, variant allele count, variant allele frequency, variant allele quality, and p-value of SNV calls. The numbers beside each boxplot indicate the mean±standard deviation of the factor values in the group of SNV calls. T-test p-values are shown.

To have an overall picture, we first pooled all SNV calls into two groups, reproduced and not-reproduced, and compared the values of those technical factors in the two groups. As expected, the mean values of coverage, variant allele count, variant allele frequency, variant allele quality, and SNV call p-values were all highly significantly higher in reproduced SNV calls than that in not-reproduced SNV calls (Fig 2). However, the high standard deviation in each group indicated large spread of the factor values in both reproduced and not-reproduced groups. To examine the precise effect of the factors and to identify their potential thresholds, we categorized each factor and studied their effect in detail below.

#### Nucleotide substitution types

**Fig 3:**
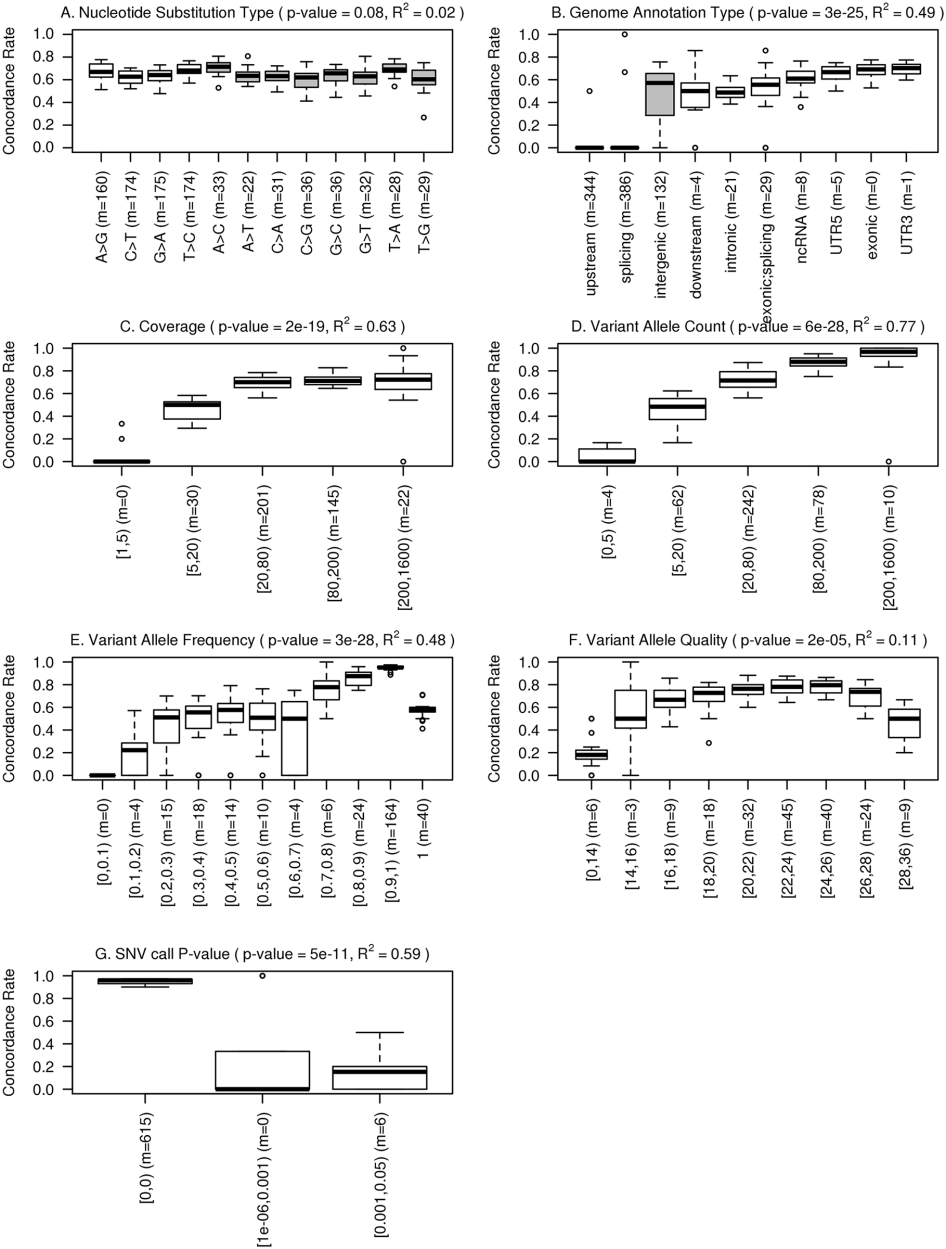
Boxplots of SNV call concordance between replicated samples by different factors, including (A)_nucleotide substitution type (gray-shaded boxes = transversions, open boxes = transitions), (B) genome annotation type, (C) coverage, (D) variant allele account, (E) variant allele frequency, (F) variant allele quality, and (G)) SNV call p-value. The numbers in parenthesis (m) represents the median of the numbers of SNVs per replicated sample that were counted in a given category. ANOVA test p-values are shown. Coefficient of determination (R^2^), indicating the proportion of the total variation of concordance rate that is explained by the factor alone, is shown for each factor.

There was no statistically significant difference in concordance rates between nucleotide transitions (A>G, C>T, G>A and T>C) or transversions (A>C, A>T, C>A, C>G, G>C, G>T, T>A and T>G) by Wilcoxon rank sum test (Fig 3A). However, there were significantly more SNVs that represented transitions (range 111–271; medians of 169–184 per replicated sample per nucleotide substitution type) compared to transversions (range 16–62; medians of 23–38 per replicated sample per nucleotide substitution type) (see also S1 Fig).

#### Genome annotation types

The SNVs were annotated by location in the genome as exonic, intronic, 5’- and 3’-UTR, intergenic, downstream, upstream, exonic/splicing, splicing, or noncoding RNA (ncRNA). As expected from targeted sequencing of the kinome, intergenic, upstream, downstream, exonic/splicing, and splicing regions had overall low coverage and few SNVs fell into these regions (0–19 per sample, S1 Fig). Concordance rates for these regions had large variance. Among the rest of the annotation types, the median concordance rate of the intronic regions (0.5) was significantly lower (Wilcoxon rank sum test p-values = 1.2x10^-7^–6.2x10^-6^) than the median concordance rates of the exonic, 3’-UTR and 5’-UTR regions, respectively (each around 0.7) (Fig 3B). Concordance rates were similar for synonymous and non-synonymous exonic SNVs (S2 Fig).

#### Coverage

We examined concordance by coverage depth in five empirical coverage bins, including “1-4x", "5-19x", "20-79x", "80-199x", "≥200x". Concordance rate increased significantly with coverage until it reached the 20-79x coverage level (Wilcoxon p-value = 10^-8^–10^-4^) (Fig 3C). Further increase in coverage did not significantly increased concordance rates above around 70% even at high levels of coverage (≥200x).

Since the target enrichment kit was designed to capture exons, we hypothesized that the difference in concordance rates between exonic and intronic regions is caused by differences in coverage depths (i.e. higher coverage of exonic regions). The coverage level of SNVs in exonic regions was significantly higher compared to SNVs in intronic regions (average 65x *vs* 44x, Wilcoxon rank sum test p-value<2.2x10^-16^). Next, we examined concordance rates within exonic and intronic regions while controlling for coverage depth. The difference in concordance rates between intronic and exonic regions, and between intronic and 3’- and 5’-UTR regions, were much smaller within each stratified coverage level compared to concordance rates calculated across all coverage levels which confirms that coverage depth is an important driver of concordance rates (S3 Fig).

To further assess the impact of coverage depth on concordance of SNV calls, we filtered out all positions with coverage <20x in either or both paired replicates and re-calculated the concordance rates for each pair. This coverage-based filtering significantly improved concordance for all SNVs (median of 73.1%, p-value = 0.006) but had lesser impact on the concordance rates for HFI SNVs (median of 66.7%) due to the much larger sample-to-sample variation in difference in concordance rate before and after filtering.

In order to identify pre-analytical variables that may influence coverage depth, we examined the association between coverage value and (i) length of DNA storage (5–71 months), (ii) origin of tissue sample (metastatic versus primary cancer), (iii) purity (OD 260/280 ranged between 1.5–2.38) and (iv) concentration (17.9–274.4 ng/μl) of the input DNA above the a priory defined QC thresholds. No significant association was detected. On the other hand, the total number of reads was significantly positively associated with greater average coverage (S4 Fig).

#### Variant allele count (VAC)

VAC is the number of reads of the most abundant non-reference allele at a position. The values of VAC correlated strongly with coverage (Spearman correlation coefficient of 0.86) and also showed a strong positive relationship with concordance rate. With the increase of VAC, the concordance rate increased steadily without a plateau. The median concordance rates reached 100% in the last two bins of variant allele count (≥158x) (Fig 3D).

#### Variant allele frequency (VAF)

VAF is the proportion of the variant allele out of all allele reads at a given position. From its distribution in all SNV positions, the variant allele frequency peaked at around 1. There was another much lower peak at VAF around 0.5. The concordance rate also increased as the VAF increased (Fig 3E). There seemed to be a significant increase in the concordance rate for positions with a VAF higher than or equal to 0.7 compared to those that below 0.7 (Wilcoxon rank sum test p-values = 10^-7^-10^-4^).

#### Variant allele quality (VAQ)

VAQ is the mean of the individual quality values of all variant allele reads at a given position. The VAQ values ranged from 0 to 35 across all positions. The concordance rate increased with the increase of variant allele quality (except for the highest quality category, VAQ ≥ 28) (Fig 3F). The concordance rate was significantly higher in positions with quality range of 20–28 than those with a quality value < 20 (Wilcoxon rank sum test p-values = 10^-7^-0.01).

#### P-value for each SNV call by Bioscope

The majority (91%) of the p-values at called SNV positions were 0 (i.e. highly significant). Only 2% of the p-values were 1, and 7% were between 0 and 1. The concordance rates for positions that had SNV-call p-value of 0 in both replicated samples were very high (mean concordance rate = 0.95, standard deviation = 0.03) and significantly greater than positions with higher p-values (Fig 3G). However, the median number of SNV positions in the p = 0 category was only 615, while the average number of SNVs per replicated sample was 1007. Almost 40% of SNV positions per sample were filtered out because they had p-value of 0 in one sample and non-zero in the replicated sample.

#### GC content

One of the potentially important factors in sequencing was GC content. The GC content in the targeted kinome region in this study had a loosely bimodal distribution separated at 50% (S5 Fig). The coverage depths in low-GC content regions were significantly higher than those in high-GC content regions (mean coverage of 116x vs. 39x, p-value < 10^-16^). The concordance rates in the low-GC content regions were significantly higher than those in the high-GC content regions (mean concordance rate of 0.55 vs. 0.40, p-value = 1.5x10^-6^, S5 Fig).

#### Variation explained by factors

Most of the factors we considered indeed had a significant impact on concordance rate of SNV calls. To quantify this impact, coefficient of determination (R^2^) was used to measure the proportion of the total variation of concordance rate that was explained by each factor alone (Fig 3). VAC and coverage alone explained most of the variation (77% and 63%), followed by p-value of SNV call, VAF, and genome annotation type (59%, 48%, and 49%). VAQ explained the least of the variation among the significant-impact factors (11%).

### Relative importance of different factors

Since five of the above factors including coverage, variant allele count, variant allele frequency, variant allele quality, and SNV call p-value each significantly associated with variant call concordance rate, we quantitatively compared the relative importance of these factors and their prediction ability for concordance rate.

**Fig 4:**
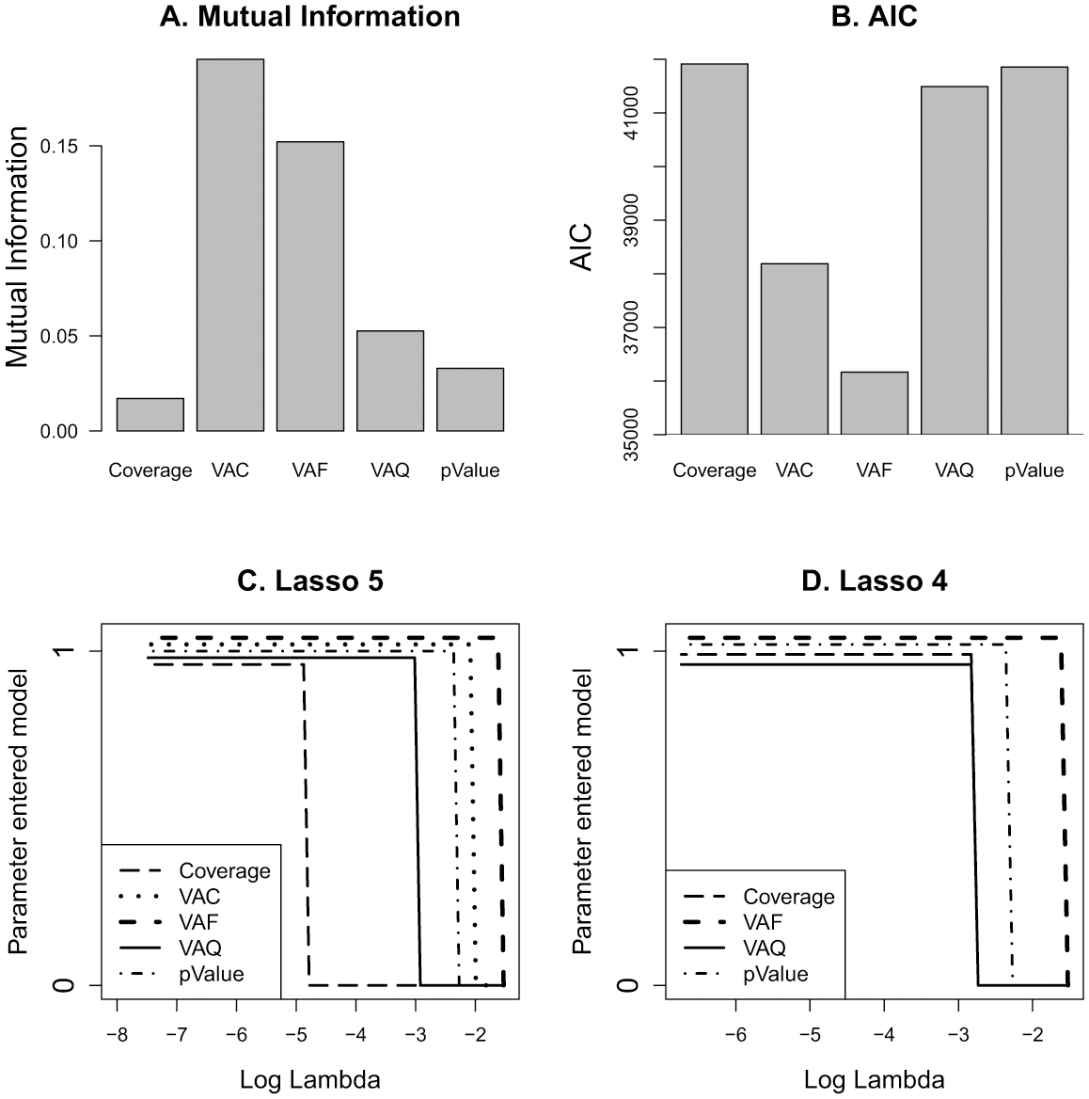
Comparisons between the relative importance of the 5 different variables in determining reproducibility of SNV calls. Importance was assessed using mutual information value (A), Akaike information criterion (B), and Lasso regression methods (C, D). On panels C and D, the y-axis indicates whether a factor is in the model (y = 1) or not (y = 0). VAC = variant allele count, VAF = variant allele frequency, VAQ = variant allele quality and p-value refers to SNP call p-value generated by BioScope.

First, we examined correlation between these 5 factors. Only coverage depth and variant allele count showed a strong linear correlation (S6 Fig). Checking the association of these factors with concordance status (concordant or discordant) was done by using mutual information (the factors were treated as discrete variables) and logistic regression (the factors were treated as continuous variables). Mutual information measures the dependence between two variables, and larger values of mutual information indicate more dependency between the variables [9]. VAC and VAF showed much larger mutual information with concordance status than the other three factors (Fig 4A). The Akaike information criterion (AIC) is often used in model selection, and models with smaller values of AIC are preferred. VAC and VAF were again the two factors with the smallest AIC values while all other factors showed comparable but larger values (Fig 4B).

We also performed Lasso model selection [10] to identify factors that are the most associated with concordance status (regular least squares method was not able to separate the factors because they were all highly associated with concordance status). With penalty value λ goes from large to small, the most significant variable enters the model first, and the least significant variable enters the model last. Fig 4C shows that with the decreasing of λ from right to left, VAF entered the model first, followed by VAC, SNV-call p-value, variant allele quality and coverage. Coverage may have entered the model later than all other factors because of its close correlation with VAC. This is illustrated by Fig 4D, which shows the same Lasso plot after removing VAC from the variables. We see that factor VAF entered the model first, followed by SNV-call p-value, and variant allele quality and coverage entered the model at the same time. In summary, all 3 methods identified VAF and VAC as the most consistently associated variables with variant call concordance status.

### Effect of factor filtering and duplicates removal

In order to verify our results and identify possible criteria for reproducible SNV calls, we generated SNV calls using another popular variant calling algorithm, VarScan2 [7]. The unfiltered SNV calls were generated using default parameters except for requiring p-value less than 0.05. Filtering criteria that were based on the pooled reproduced SNVs (Fig 2) included coverage (min-coverage) of 78, VAC (min-reads2) of 45, VAF (min-var-freq) of 0.6, and VAQ (min-avg-qual) of 22. Filtering criteria that were based on the categorized concordance analysis (Fig 3) included coverage of 20, VAC of 20, VAF of 0.3, and VAQ of 20.

The mean concordance rate of the unfiltered SNV calls was 22.5%, which significantly increased to 45.1% after filtering by criteria based on the pooled reproduced SNV calls, which further significantly increased to 52.9% after filtering by criteria based on the categorized concordant SNV calls (p-values < 10^-10^, Table 3). This result verified that these factors provide effective filtering criteria for more reliable SNV predictions.

**Table 3:** Concordance rates of SNVs called by VarScan2 program between replicated samples and after removal of duplicated reads.

	Unfiltered mean (sd) %	Filtered by criteria based on pooled SNV calls mean (sd) %	Filtered by criteria based on categorized SNV calls mean (sd) %
VarScan2	22.5 (7.7)	45.1 (8.9)^a^	52.9 (11.6) ^a^ ^,^ ^b^
Remove dup	27.6 (8.3)	56.5 (8.3) ^a^	58.9 (12.7) ^a^

Column one (Unfiltered) contains all SNV calls generated using default parameters except for requiring p-value less than 0.05. Column two contains SNV calls that were filtered based on results from pooled SNV calls as shown in Fig 2. Column three contains SNV calls that were filtered based on results from categorized SNV calls as shown in Fig 3. Row two (Remove dup) shows the same variables after removal of duplicated reads.

^a^ Significantly different from the concordance rates of unfiltered SNV calls (p-value < 10^-10^).

^b^ Significantly different from the concordance rates of filtered by criteria based on pooled SNV calls (p-value = 0.013).

To assess the potential impact of duplicated reads that were likely caused by PCR amplification, we removed duplicated reads and repeated the above analysis. Removal of duplicates alone increased the mean concordance rate of unfiltered SNV calls from 22.5% to 27.6%, significant (p-value = 0.03) but not substantial. For the SNV calls that were filtered by the pooled criteria, the concordance rate significantly increased from 45.1% to 56.5% after removal of duplicated reads (p-value = 3x10^-5^). For the SNV calls that were filtered by the categorized criteria, the increase in concordance rates after removal of duplicates was not significant, but also showed an increasing trend (52.9% increased to 58.9%, Table 3). In summary, removal of duplicates combined with filtering significantly and substantially increased the concordance rates.

## Discussion

The overall concordance rate for all nucleotides across the entire sequenced region was high (>99.99%) but the concordance rate between variant calls was substantially lower, 54.3% -75.5%. We also observed substantial variability in basic sequencing metrics such as the number of total reads and depth of coverage in a particular nucleotide location in repeat experiments. Coverage depth had an important correlation with reproducibility of variant calls. Since the number of total reads directly influences coverage, it is also the most easily controlled factor in sequencing experiments that could increase reproducibility. Call concordance increased significantly as coverage reached ≥20x, however, it did not increase beyond 80% even at very high coverage levels indicating that other factors also play important roles in influencing the reproducibility of variant calls. Variant allele count, variant allele frequency, variant allele quality, and the p-value of the SNV-call also correlated significantly with concordance rates and variant allele count and variant allele frequency were the most important factors.

Interestingly, the concordance rates in the highest categories of variant allele frequency and variant allele quality dropped despite a clear and significant association between higher values and greater concordance (Fig 3E–3F). We hypothesize that PCR artifacts may cause this during library preparation; a small portion of genomic DNA fragments may be randomly amplified to much higher level than other fragments during the PCR reaction resulting in very high allele frequency and allele quality for these sequences but low reproducibility during repeat library preparation [11]. Because of the enrichment step, deep sequencing of the targeted regions possibly have many duplicated reads. Our study showed that removal of duplicated reads combined with filtering by the key factors significantly and substantially increased the concordance rates.

Although it was previously shown that the SOLiD system was less prone to GC-content based bias compared to other techniques [12], our study showed significantly lower coverage and lower concordance rate in high-GC content regions. However, since in general we cannot control GC content in an experiment, it was not used as a filtering factor in our study.

Several previous studies examined concordance between known SNVs detection by next generation sequencing and by SNP arrays [2, 13]. These studies showed high sensitivity of sequencing to detect the known variants in a given sample. Our observations are consistent with these reports; we also noted high overall sequencing concordance in replicate experiments that translate into high sensitivity to detect specific variants. However, we also show that concordance rates for novel variant calls are much lower. This is in contrast with one previous small study (n = 4) that addressed a similar question and suggested that novel SNV detection concordance rates are ≥ 95% in biological replicates [14]. It is not clear from the methods section of that paper how the replications were exactly performed. In our study, we replicated every step in the sequencing procedure starting from aliquots of the same genomic DNA and the replicates were performed on different days that may account for the higher discordance rates.

In summary, our study suggests that even at reasonably high coverage levels variant calls using the SOLiD v4 sequencing platform and BioScope software have less than optimal reproducibility. We recognize that sequencing platform, read lengths, library type and read mapping/variant calling software may affect the reproducible variant calls. However, in our experiments, even applying the highest stringency QC metrics only yielded SNV reproducibility calls around 80%. These results suggest that global mutation differences observed in various biological tissues such as primary cancer and metastasis or different regions of the same tumor contain a substantial amount of technical noise.

## Supporting Information

S1 Fig Boxplots showing concordant and discordant SNVs by substitution and annotation. A & B: Boxplots of the number of concordant and discordant SNVs within different nucleotide substitution types. Red boxes indicate nucleotide transitions and blue boxes indicate nucleotide transversions. C & D: Boxplots of the number of concordant and discordant SNVs by genome annotation type. E: Boxplot of the average coverage within each genome annotation region per sample for all sequenced regions.
(PDF)

S2 Fig Boxplot showing the concordance rates of the synonymous and non-synonymous SNVs, respectively. (PDF)

S3 Fig Boxplots of concordance rate of SNV call by genomic annotation type, stratified by coverage levels. Concordance rate of coverage category [1,5) is not shown due to small number of SNVs in this category.
(PDF)

S4 Fig Scatter plots of average coverage in target region versus the number of total reads per sample. The Pearson correlation coefficients (Pearson CC) and p-value of the correlation test (Pearson Correlation p-value) are as shown.
(PDF)

S5 Fig GC content. A: Histogram and density plot showing the empirical distribution of GC-content in the targeted Kinome region in this study. B: Coverage depths were significantly higher in GC-low regions than those in GC-high regions. C: The concordance rates in GC-low regions were significantly higher than those in the GC-high regions.
(PDF)

S6 Fig Pairwise correlation between coverage, variant allele count (VAC), variant allele frequency (VAF), variant allele quality (VAQ) value, and SNV-call p-values. The panels below the diagonal line show the pairwise smoothed scatter plots between the factors for all the SNV positions from the replicate experiments. A locally weighted smooth regression (LOWESS) line is also shown for each scatter plot. The panels above the diagonal show the values of pairwise Spearman correlation coefficients.
(JPG)

S1 Table Distribution of the replicated samples in different sequencing batches ordered by the dates in which each multiplexed library was sequenced. (DOCX)
