# Supplementary figures and images for: Reproducibility of Variant Calls in Replicate Next Generation Sequencing Experiments
Source: PLoS One. 2015 Jul 2;10(7):e0119230. doi: 10.1371/journal.pone.0119230 (PMC4489803; doi:10.1371/journal.pone.0119230)

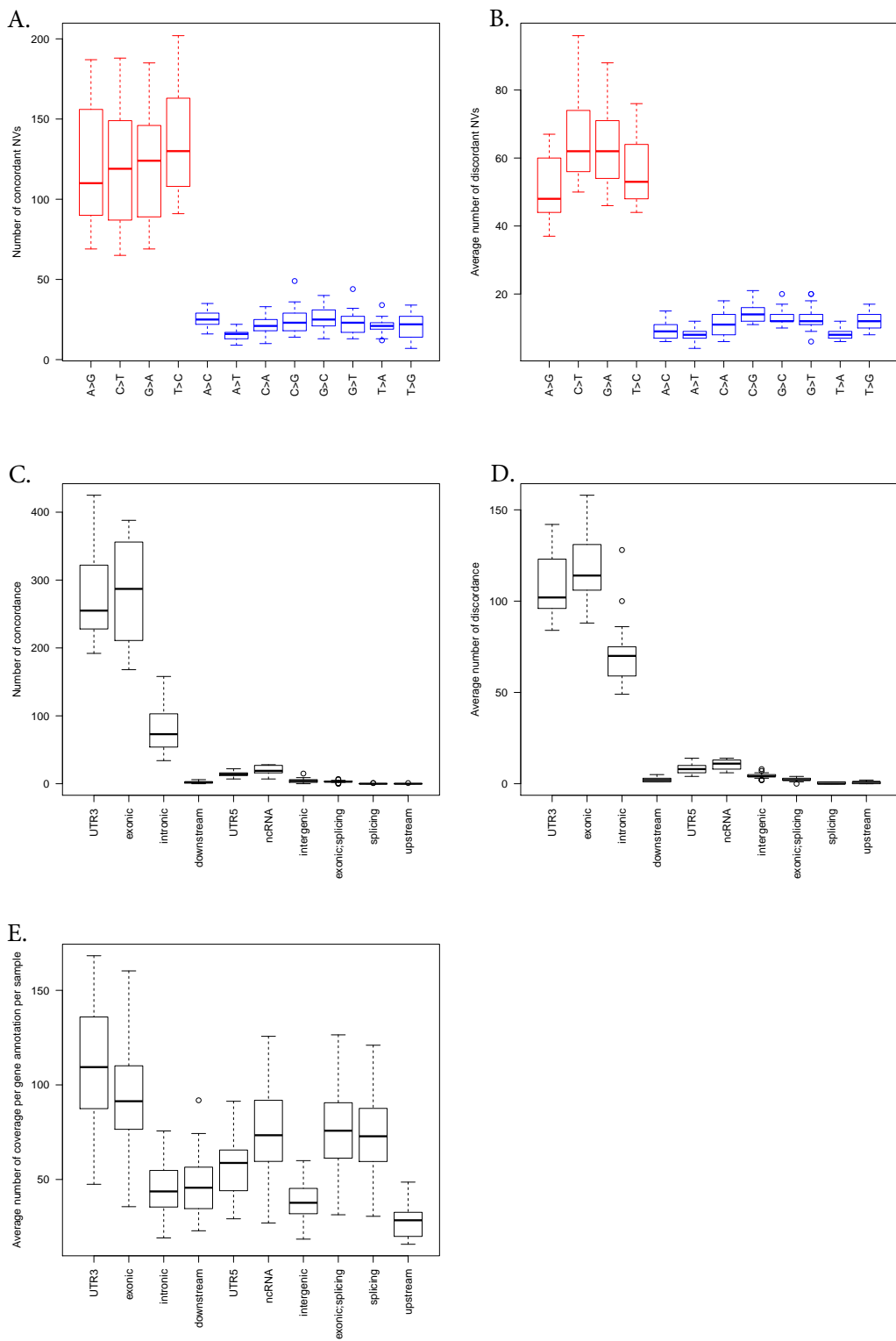

Supplement: S1 Fig — A & B: Boxplots of the number of concordant and discordant SNVs within different nucleotide substitution types. Red boxes indicate nucleotide transitions and blue boxes indicate nucleotide transversions. C & D: Boxplots of the number of concordant and discordant SNVs by genome annotation type. E: Boxplot of the average coverage within each genome annotation region per sample for all sequenced regions. (PDF) [file pone.0119230.s001.pdf]

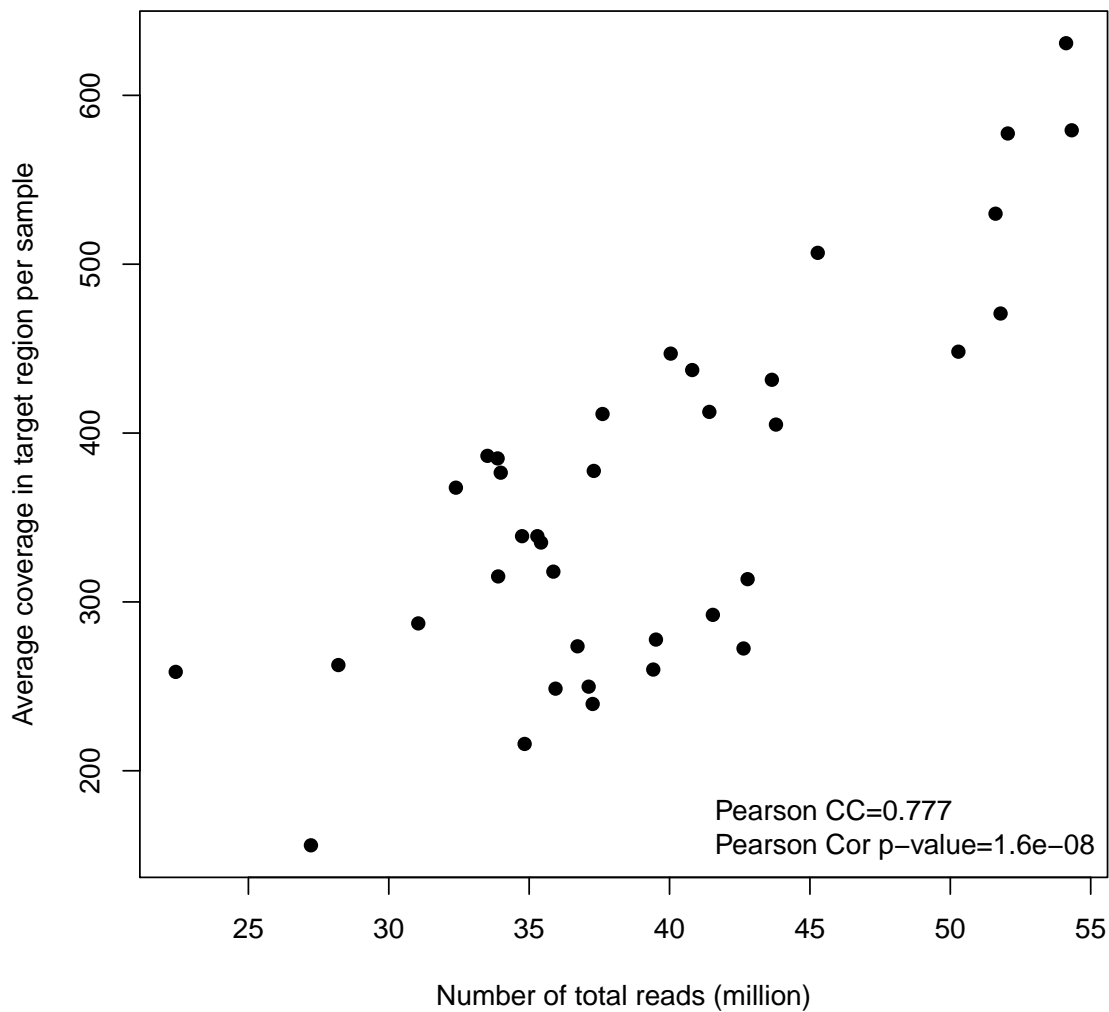

Supplement: S4 Fig — The Pearson correlation coefficients (Pearson CC) and p-value of the correlation test (Pearson Correlation p-value) are as shown. (PDF) [file pone.0119230.s004.pdf]

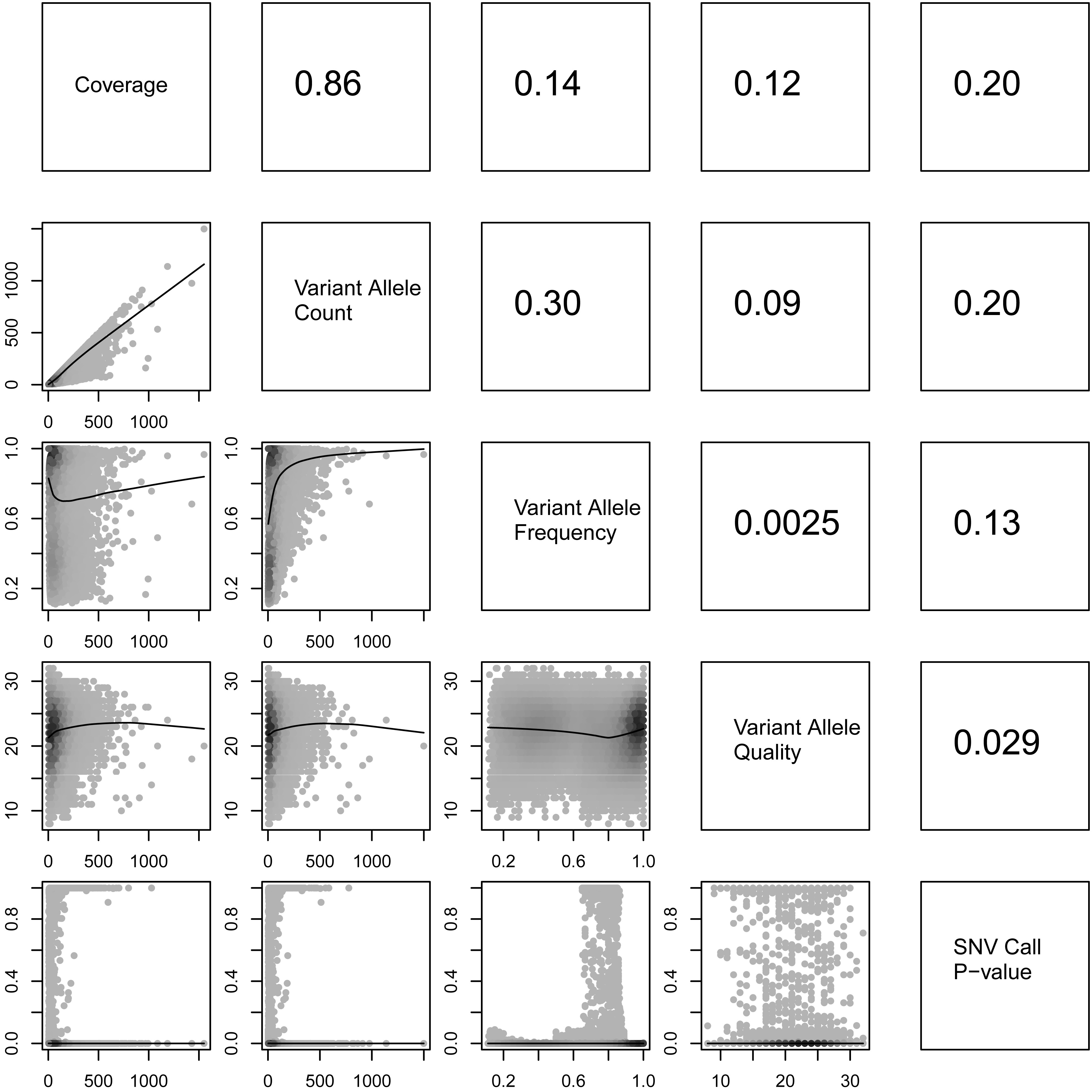

Supplement: S6 Fig — The panels below the diagonal line show the pairwise smoothed scatter plots between the factors for all the SNV positions from the replicate experiments. A locally weighted smooth regression (LOWESS) line is also shown for each scatter plot. The panels above the diagonal show the values of pairwise Spearman correlation coefficients. (JPG) [file pone.0119230.s006.jpg]
