# Supplementary material for: Reproducibility of Variant Calls in Replicate Next Generation Sequencing Experiments
Source: PLoS One. 2015 Jul 2;10(7):e0119230. doi: 10.1371/journal.pone.0119230 (PMC4489803; doi:10.1371/journal.pone.0119230)
Supplement: S2 Fig — (PDF) [file pone.0119230.s002.pdf]

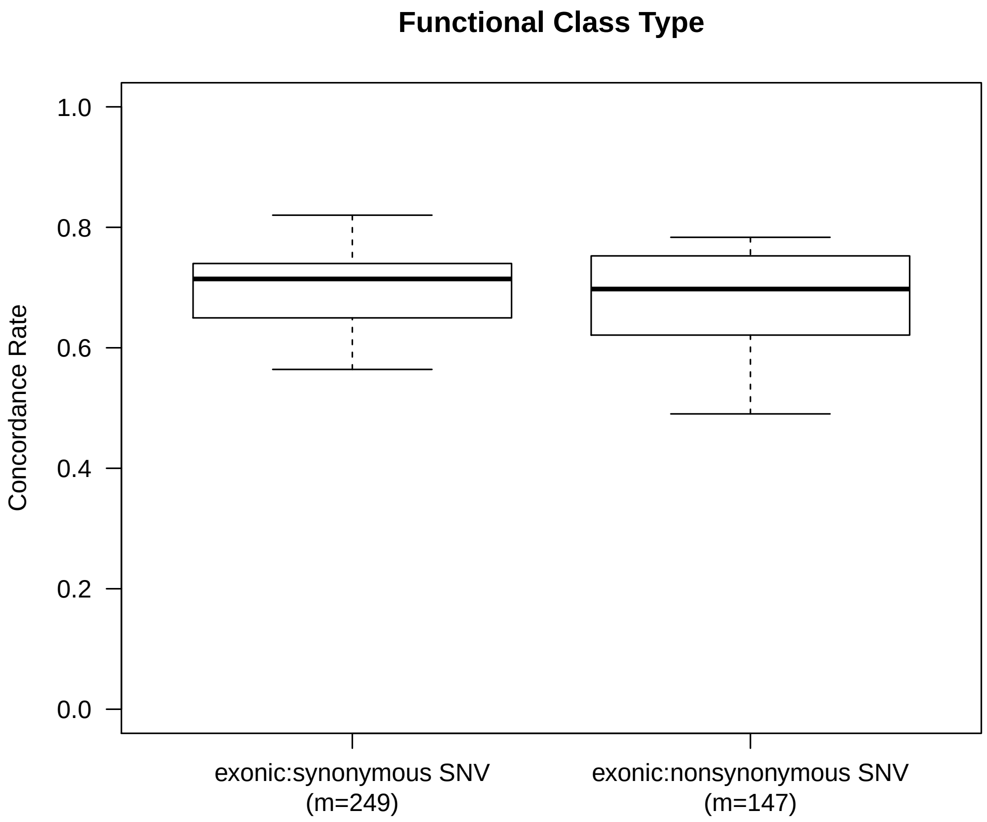

Supplementary Figure 2: Boxplot showing the concordance rates of the synonymous and non-synonymous SNVs, respectively.
