# Supplementary material for: Reproducibility of Variant Calls in Replicate Next Generation Sequencing Experiments
Source: PLoS One. 2015 Jul 2;10(7):e0119230. doi: 10.1371/journal.pone.0119230 (PMC4489803; doi:10.1371/journal.pone.0119230)
Supplement: S3 Fig — Concordance rate of coverage category [1,5) is not shown due to small number of SNVs in this category. (PDF) [file pone.0119230.s003.pdf]

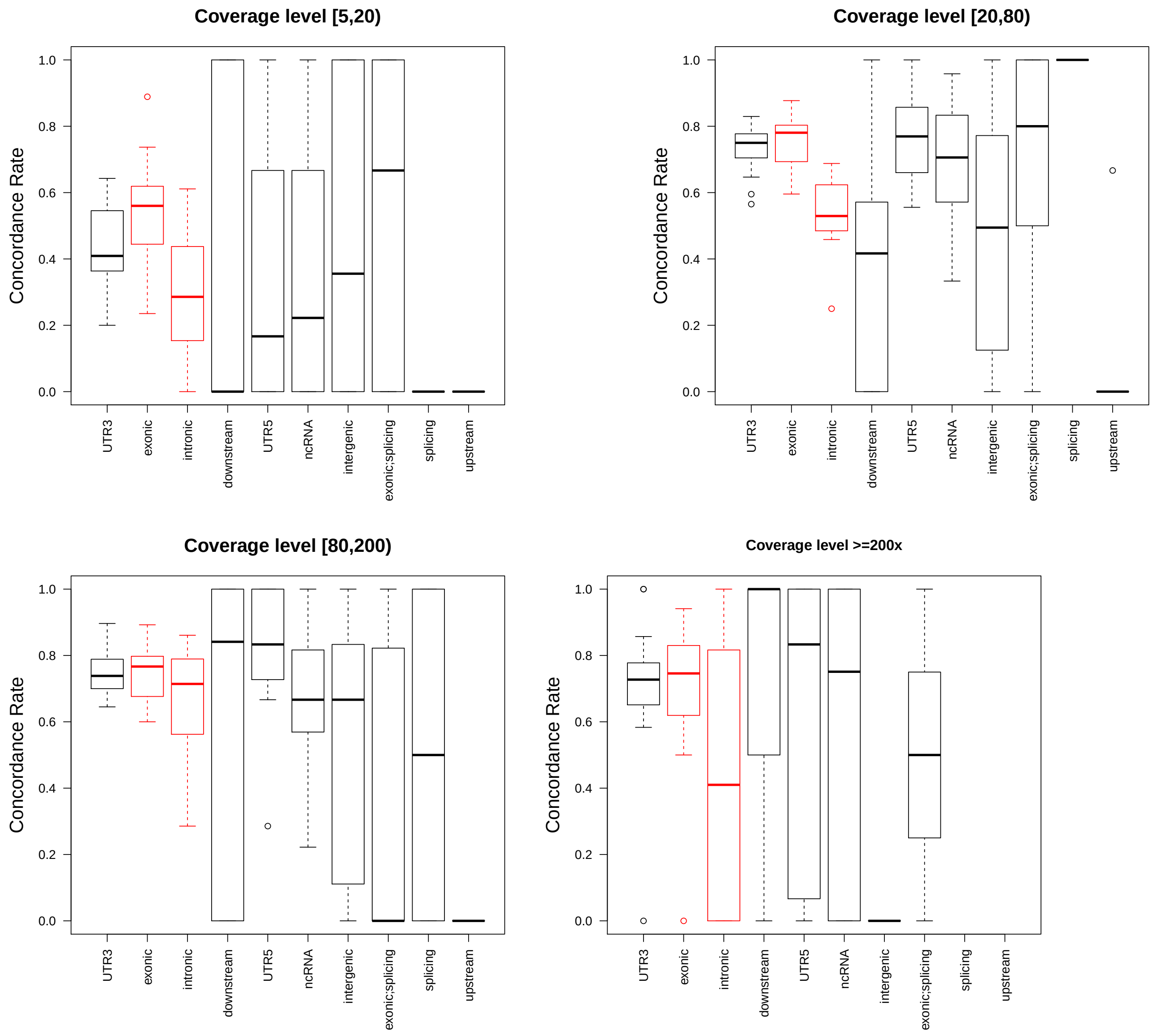

Coverage level [5,20)
Concordance Rate
0.0
0.2
0.4
0.6
0.8
1.0
UTR3
exonic
intronic
downstream
UTR5
ncRNA
intergenic
exonic;splicing
splicing
upstream
Coverage level [20,80)
Concordance Rate
0.0
0.2
0.4
0.6
0.8
1.0
UTR3
exonic
intronic
downstream
UTR5
ncRNA
intergenic
exonic;splicing
splicing
upstream
Coverage level [80,200)
Concordance Rate
0.0
0.2
0.4
0.6
0.8
1.0
UTR3
exonic
intronic
downstream
UTR5
ncRNA
intergenic
exonic;splicing
splicing
upstream
Coverage level >=200x
Concordance Rate
0.0
0.2
0.4
0.6
0.8
1.0
UTR3
exonic
intronic
downstream
UTR5
ncRNA
intergenic
exonic;splicing
splicing
upstream
