# Supplementary material for: Reproducibility of Variant Calls in Replicate Next Generation Sequencing Experiments
Source: PLoS One. 2015 Jul 2;10(7):e0119230. doi: 10.1371/journal.pone.0119230 (PMC4489803; doi:10.1371/journal.pone.0119230)
Supplement: S5 Fig — A: Histogram and density plot showing the empirical distribution of GC-content in the targeted Kinome region in this study. B: Coverage depths were significantly higher in GC-low regions than those in GC-high regions. C: The concordance rates in GC-low regions were significantly higher than those in the GC-high regions. (PDF) [file pone.0119230.s005.pdf]

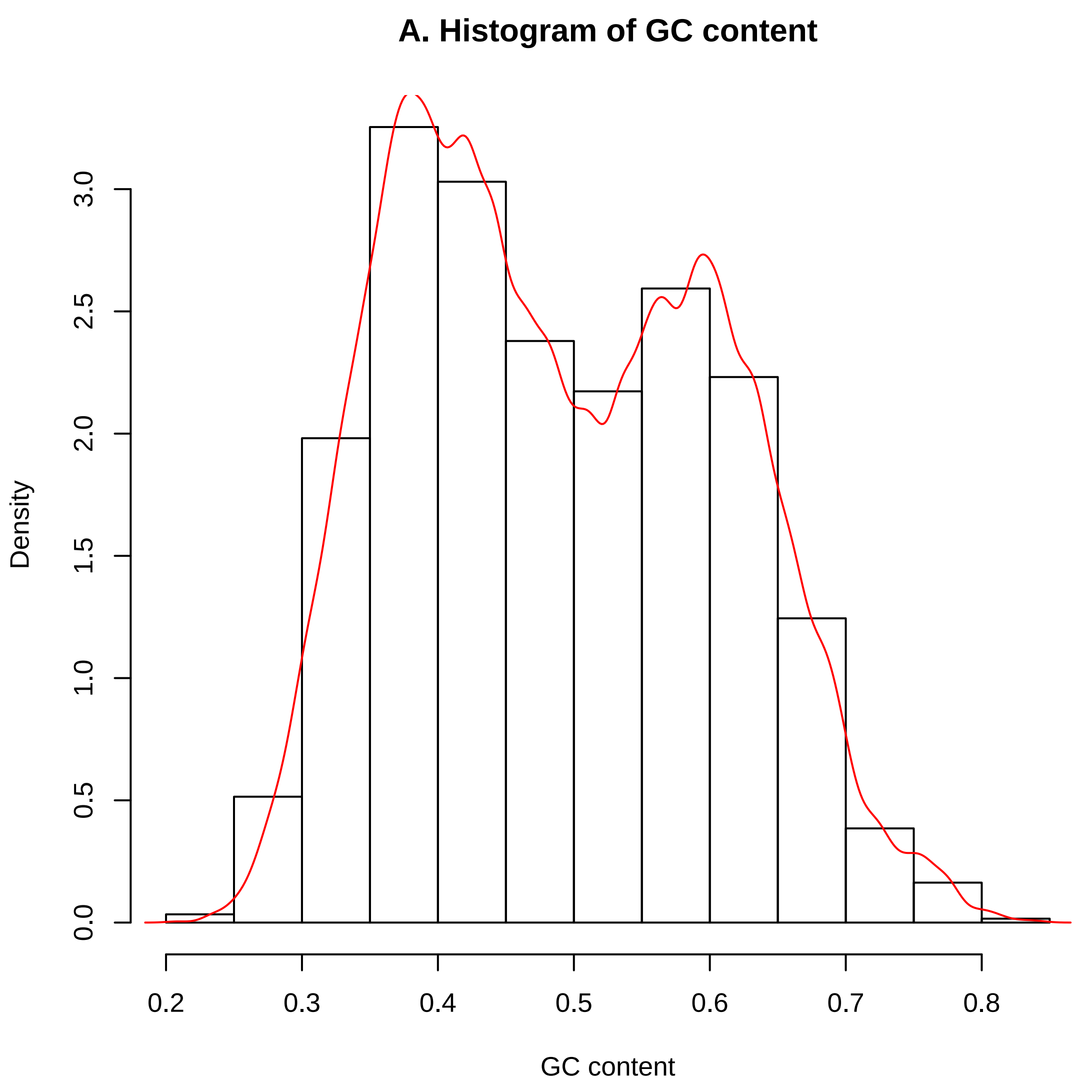

A. Histogram of GC content
Density
0.0
0.5
1.0
1.5
2.0
2.5
3.0
0.2
0.3
0.4
0.5
0.6
0.7
0.8
GC content

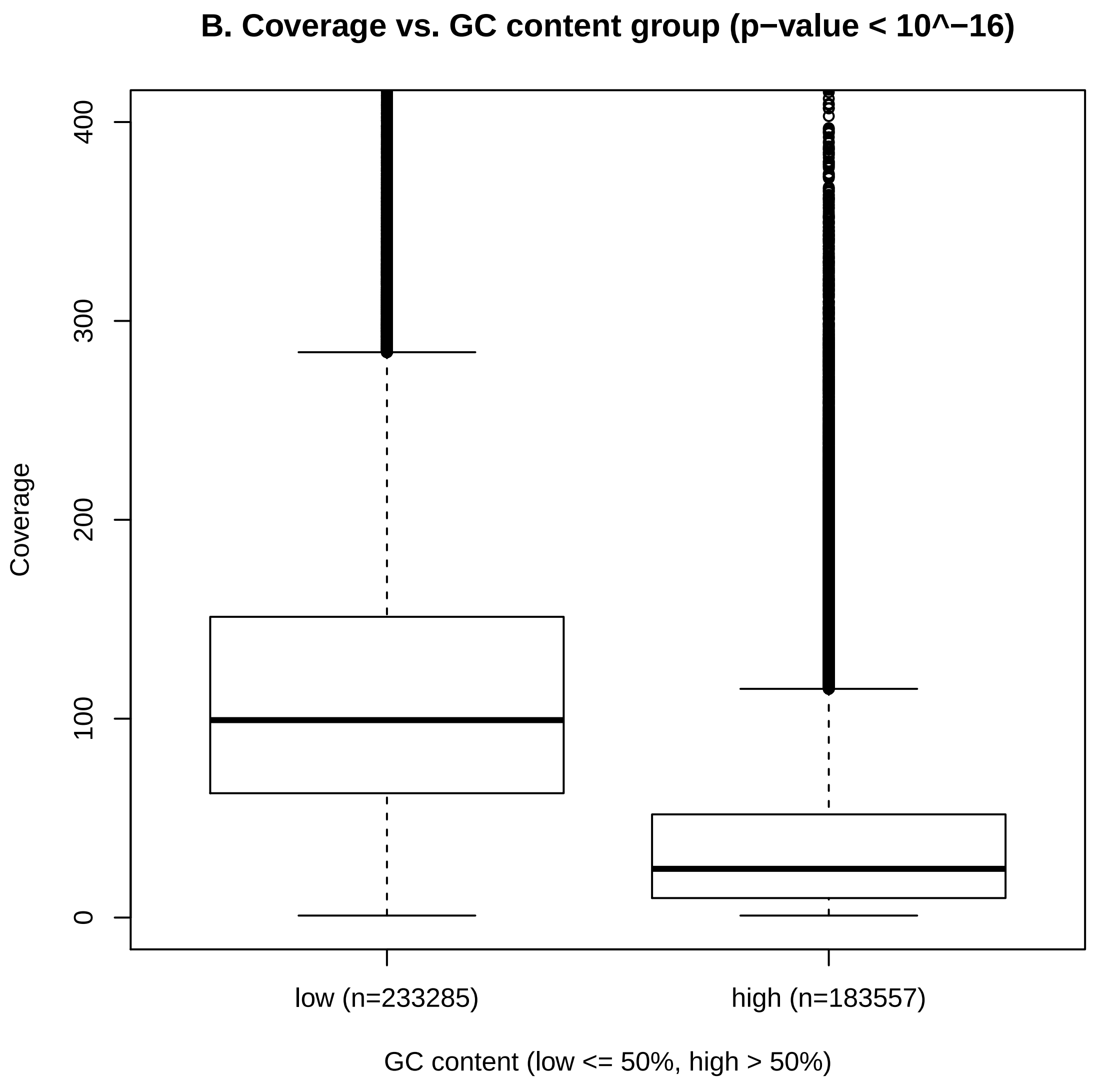

B. Coverage vs. GC content group (p−value < 10^−16)
Coverage
0
100
200
300
400
low (n=233285)
high (n=183557)
GC content (low <= 50%, high > 50%)

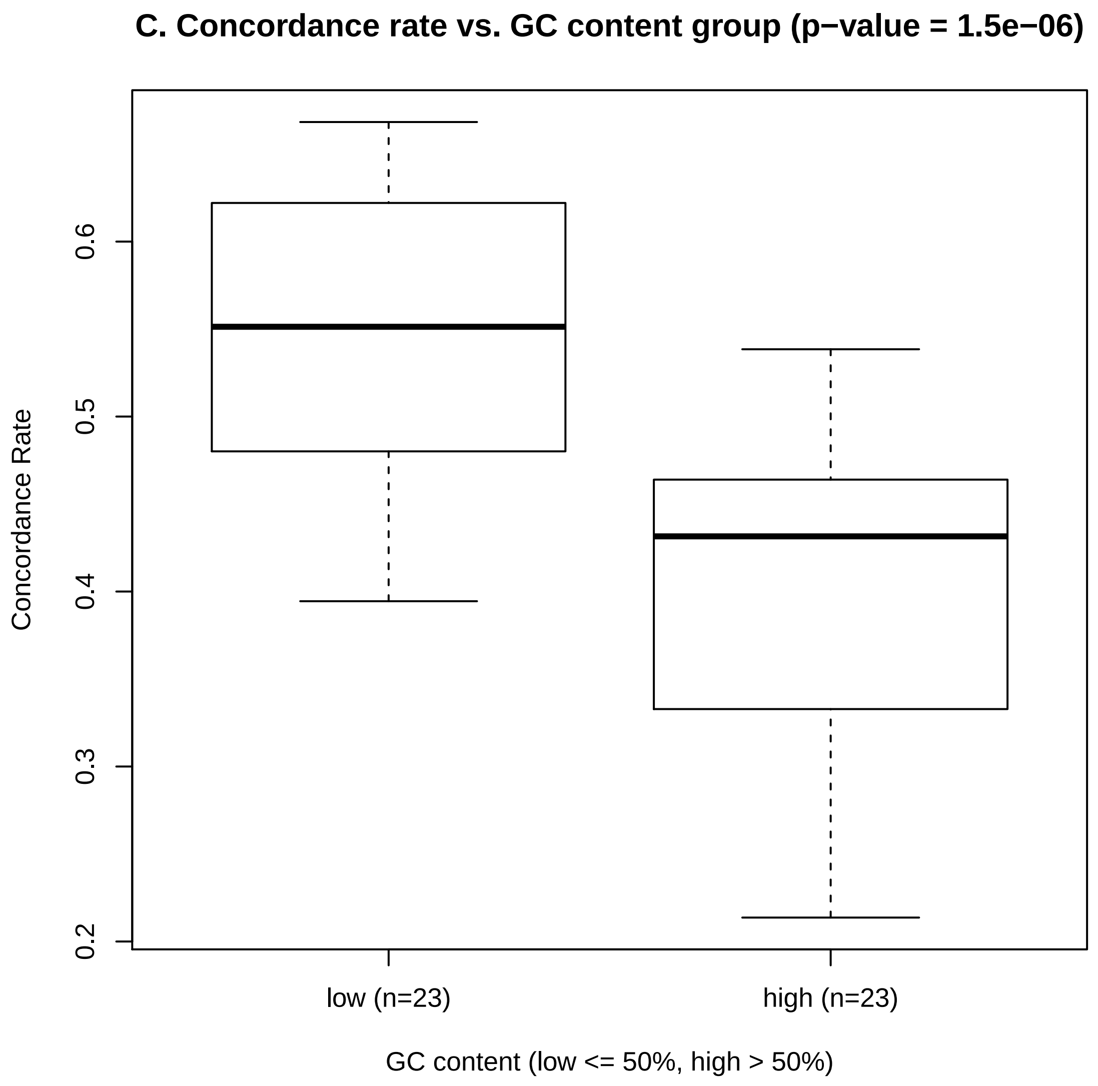

C. Concordance rate vs. GC content group (p−value = 1.5e−06)
Concordance Rate
0.2
0.3
0.4
0.5
0.6
low (n=23)
high (n=23)
GC content (low <= 50%, high > 50%)
