# Supplementary material for: Reproducibility of Variant Calls in Replicate Next Generation Sequencing Experiments
Source: PLoS One. 2015 Jul 2;10(7):e0119230. doi: 10.1371/journal.pone.0119230 (PMC4489803; doi:10.1371/journal.pone.0119230)
Supplement: S1 Table — (DOCX) [file pone.0119230.s007.docx]

**S1 Table. Distribution of the replicated samples in different sequencing batches (T1-T7) ordered by the dates in which each multiplexed library was sequenced.** (R1=sequencing experiment 1, R2=sequencing experiment 2, R3=sequencing experiment 3)

| Sample ID | T1 (2010-12-03) | T2 (2010-12-13) | T3 (2010-12-13) | T4 (2011-02-15) | T5 (2011-03-18) | T6 (2011-03-18) | T7 (2011-04-06) |
| --- | --- | --- | --- | --- | --- | --- | --- |
| 294 |  |  | R1 |  | R2 |  |  |
| 339 |  |  |  | R1 |  | R2 |  |
| 494 |  |  | R1 |  |  | R2 |  |
| 506 | R1 |  |  | R2 |  |  |  |
| 512 |  |  |  | R1 | R2 |  |  |
| 524 |  |  |  |  | R1 |  | R2 |
| 571 | R1 |  |  | R2 | R3 |  |  |
| 647 |  |  |  | R1 | R2 |  |  |
| 658 |  |  | R1 |  | R2 |  | R3 |
| 763 |  |  |  | R1 |  | R2 |  |
| 792 | R1 |  |  | R2 |  |  |  |
| LP16 | R1 | R2 |  |  |  |  |  |
| LP40 | R1 | R2 |  |  |  |  |  |
| LP52 |  | R1 |  | R2 |  |  |  |
| LP63 | R1 | R2 | R3 |  |  |  |  |
| LP9 | R1 | R2 |  |  |  |  |  |
| M15 |  |  |  | R1 |  | R2 |  |
